# Radiotherapy for glioblastoma in the elderly: A protocol for systematic review and meta-analysis

**DOI:** 10.1097/MD.0000000000023890

**Published:** 2020-12-24

**Authors:** Puxin Huang, Liqiang Li, Juntang Qiao, Xiang Li, Peng Zhang

**Affiliations:** a Neurosurgery Department, XiGu District of Lanzhou City People's Hospital; b Neurosurgery Department, Gansu Gem Flower Hospital, No. 733, Fuli Road, Xigu District, Lanzhou city, Gansu Province, China; .

**Keywords:** glioblastoma, radiotherapy, systematic review

## Abstract

**Background:**

Glioblastoma is an aggressive form of brain cancer with significant morbidity and mortality. This study aims to determine the radiotherapy for treatment of elderly people with diagnosed glioblastoma.

**Method:**

This study adheres to the Preferred Reporting Items for Systematic Reviews and Meta-analysis for Protocols. Chinese electronic Database (CBM, Wanfang, and CNKI) and international electronic databases (PubMed, Embase, Cochrane Library, and Web of Science) will be searched for all relevant published articles, with no restrictions on the year of publication or language. Study selection, data collection, and assessment of study bias will be conducted independently by a pair of independent reviewers. The Cochrane Risk of bias (ROB) tool will be used for the risk of bias assessment. The Grading of Recommendations Assessment Development and Evaluation (GRADE) system will be used to assess the quality of evidence. The statistical analysis of this meta-analysis will be calculated by Review manager version 5.3.

**Results:**

The results of this study will be published in a peer-reviewed journal.

**Conclusion:**

The findings of this review will to provide high-level evidence in terms of the benefits and harms of radiotherapy in people with glioblastoma to provide meaningful conclusions for clinical practice and further research.

**Trial registration:**

This study protocol was registered in open Science framework (OSF), (Registration DOI: 10.17605/OSF.IO/A6BCS).

## 1 Introduction

Glioblastomas (GBMs) are highly aggressive brain tumors of the central nervous system, and the most malignant type of astrocytic tumor with a poor prognosis.^[1]^ About 5 of every 100 patients with glioblastoma survived for 5 years after diagnosis,^[2]^ and the median overall survival (OS) of patients with GBM remains only 15 months.^[3]^ GBM is characterized by the progressive loss of neurologic function, including seizures, loss of motor and communication skills, and changes in cognitive ability and personality.^[4]^ The updated WHO classification from 2016^[1]^ separates 2 major types of glioblastoma (primary and secondary) based on mutations in the isocitrate dehydrogenase (IDH) 1 or 2 genes. Primary (IDH-wildtype) glioblastomas accounting for more than 90%,^[1]^ its incidence increases with age, peaking in the 74 to 84-year-old age group.^[2]^ This review thus is focused on the common IDH-wildtype tumors. GBM has a serious impact on people's quality of life and it is associated with high economic costs for patients. It is reported that the direct health care costs for the management of malignant gliomas have been estimated at USD 32,764 per patient.^[5]^

According to the current medical technology, GBM cannot be completely cured. The natural history of the disease is that patients will relapse after treatment and it will ultimately be a fatal condition.^[1]^ However, patients with glioblastoma will live longer if they get active treatment.^[6]^ The “standard of care” of treatment for patients with GBM consists of maximal surgery resection followed by radiation therapy with concomitant and adjuvant temozolomide (TMZ) chemotherapy.^[7,8]^ Surgery is an important step in the treatment of glioblastoma, the function of maximal debulking surgery is to minimize the tumor volume thereby optimizing the impact of subsequent treatment. There is evidence that surgery improves 1- and 2-year survival rates compared to biopsy alone.^[9]^ In addition, radiotherapy is commonly used in oncology. In terms of the treatment of GBM, one of the most important mechanisms of action is to promote DNA double-strand breaks in GBM tumor cells, which, if not repaired, will further cause cell death.^[10]^ Furthermore, TMZ is the most widely used chemotherapy drug for GBM patients, but it may cause off-target toxicity that requires cessation of therapy.^[11]^ There is also evidence that people with GMB who start treatment with radiation therapy and TMZ greater than 6 weeks after neurosurgery have worse OS than people who start treatment within 6 weeks.^[12]^

It is recognized that the standard approach is not always appropriate for older people with GMB. Age is an important consideration in this disease, as older age is associated with shorter survival and a higher risk of treatment-related toxicity, previous research has also demonstrated this.^[13]^ Shorter radiotherapy courses or chemotherapy alone can lead to better outcomes for the elderly than the standard course of radiotherapy.^[14]^ Considering that there is no consensus on the optimal treatment options for the glioblastoma in patients 70 years of age or older, we will perform a comprehensive systematic review to evaluate the efficacy of radiotherapy in this population.

## 2 Materials and methods

### 2.1 study registration

This protocol will be reported according to preferred reporting items for systematic review and meta-analysis protocols (PRISMA-P).^[15]^ As a part of our project, this study protocol has been registered on the open Science framework (OSF) (Registration DOI: 10.17605/OSF.IO/A6BCS).

### 2.2 Search strategy

Four international electronic databases (PubMed, Embase, Cochrane Library, and Web of Science) and 3 Chinese electronic databases [Chinese Biomedical Databases (CBM), Wanfang database, and China National Knowledge Infrastructure (CNKI)] will be searched. Our search did not restrict based on the basis of publication type, or year of publishing. The search terms and basic search strategy were as follows: (Glioblastoma OR glioblastoma∗ OR GB∗ OR astrocyt∗ OR GBM∗) AND (Aged OR Aged∗ OR old∗ or ageing∗ or geriatric∗ OR elder∗ OR “70 year∗” OR “over 70”) AND (radiotherapy OR radiotherap∗ OR radiat∗ OR radio-therap∗ OR radiation therapy OR radiation$) AND random∗. In addition, to ensure a comprehensive data collection, references of relevant reviews were searched manually to identify additional eligible studies. We will provide specific search strategy sample of PubMed and will be shown in Appendix 1.

### 2.3 Selection criteria

#### 2.3.1 Types of studies

We will include randomized controlled trials (RCTs) comparing radiotherapy with standard care or active intervention (such as chemotherapy) in elderly patients with glioblastoma.

#### 2.3.2 Types of participants

We will include studies of elderly people with diagnosed glioblastoma, either newly diagnosed or with recurrent disease. We defined “elderly” as 70 years and older.

If studies included people with other forms of glioma (we cannot extract results for from people with glioblastoma), we will include these if other forms of glioma accounted for less than 10% of the population.

#### 2.3.3 Types of interventions

We will include all available regimens of radiotherapy that were evaluated in randomized trials. The control group could receive the standard of care/active intervention, placebo, or best supportive care.

#### 2.3.4 Types of outcome measures

We will consider for evaluation any studies including at least one of the following outcomes. The primary outcome includes the OS, defined as time from randomization to death from any cause, Severe adverse events: classified according to National Cancer Institute Common Terminology Criteria for Adverse Events (NCI-CTCAE),^[16]^ including percentage of treatment-related deaths.

The secondary outcomes consist of progression-free survival (PFS), Tolerance of radiotherapy, Health-related quality of life (HRQoL) (measured with a validated quality of life questionnaire such as EQ-5D or Short Form-36 (SF-36), Cognitive impairment, as measured by an overall cognitive function score, as a change-over-time score, or reported as individual cognitive function domains, Functional impairment or disability, as measured by an overall ability score and/or as a change of ability over time score using a standardized measurement tool.

### 2.4 Study selection

Two review authors (HPX and LLQ) will independently screen the titles and abstracts based on the inclusion or exclusion criteria, and discard studies that are not applicable; however, they will initially retain studies and reviews that might include relevant data or information on studies. Two review authors (HPX and LLQ) will independently assess the retrieved abstracts and, when necessary, the full-text articles to determine which studies satisfy the inclusion criteria. Any discrepancies regarding inclusion will resolved through discussion or by consulting a third member of (ZP) the review team until consensus is reached. We will record the selection process insufficient detail to complete a PRISMA flow chart.

### 2.5 Data extraction

Two review authors (QJT and LX) will independently extract data from the selected studies using a pre-piloted standardized data extraction form in an Excel spreadsheet. We will resolve any disagreements by discussion and consultation with a third review author (ZP). We will extract the following data: Basic information of included studies (author details, publication year, country, setting; General demographic characteristics (sampling, total number of participants enrolled, age, gender, molecular type of glioblastoma); Intervention details (radiation techniques, dose, timing, duration, control intervention); and Outcomes, including of overall and PFS, serious adverse events, QoL scores, and cognitive or functional impairment. If the information present was unclear or if information was missing, we will try to obtain them through correspondence with the study authors.

### 2.6 Assessment of risk of bias in included studies

Two review authors (QJT and LX) will independently assess the risks of bias of each included study according to the Cochrane Risk of bias tool as outlined in the Cochrane Handbook for Systematic Reviews of Interventions. We will assess the risk of bias for 7 domains, including sequence generation, allocation concealment (selection bias), blinding of participants and personnel (performance bias), blinding of outcome assessors (detection bias), incomplete outcome data (attrition bias), selective outcome reporting, and other potential sources of bias. We will judge each item as being at yes (“low risk of bias”), no (“high risk of bias”), or unclear (“moderate risk of bias”). We will resolve any disagreement in bias classification by discussion to reach consensus and, if necessary, by discussion with a third review author.

### 2.7 Data synthesis and analysis

We will use Review Manager 2020 to synthesize the available data. We will calculate dichotomous outcomes as risk ratios (RRs) with 95% confidence intervals (95% CIs). For continuous outcomes (e.g., QoL scores), we will calculate mean difference (MD), or standardized mean difference (SMD) for the same continuous outcome measured with different metrics, and the 95% CIs. For time-to-event outcomes (e.g., OS), we will calculate the hazard ratio (HR) with its 95% CIs. If we are unable to perform a meta-analysis due to substantial differences between included studies, we will perform a narrative synthesis of the data.

### 2.8 Subgroup analysis and investigations of heterogeneity

We will investigate the potential sources of heterogeneity in the results for each method using subgroup analyses or meta-regression, depending on the number of studies identified and the nature of the source of heterogeneity. We will assess the clinical heterogeneity of the included studies by comparing participants’ characteristics (age, gender, extent of tumor resection, and disease duration), interventions (administration method, dosage and duration, control intervention). We will assess statistical heterogeneity among the included studies using the Chi^2^ test and the *I*^2^ statistic. When the *I*^2^ statistic value is greater than 50% (substantial heterogeneity), we will perform subgroup and sensitivity analyses to consider possible reasons for heterogeneity.

### 2.9 Sensitivity analyses

We plan to perform a sensitivity analysis restricting the analysis to studies we judge to be at low or unclear risk of bias.

### 2.10 Certainty assessment

Two trained GRADE methodologists will use Grades of Recommendation, Assessment, Development, and Evaluation (GRADE) system^[17]^ to assess the certainty (quality) of evidence associated with specific outcomes and constructed a summary of findings table. Assessment of the quality of evidence considers study methodological quality, directness of the evidence, heterogeneity of data, precision of effect estimates, and risk of publication bias.^[18]^

### 2.11 Ethics and dissemination

This study belongs to the category of systematic review and it is only a secondary analysis of the published data, so ethical approval is not applicable to this study.

## 3 Discussion

Glioblastoma is an uncommon but highly aggressive type of brain tumor. The standard of care is maximal surgical resection followed by chemoradiotherapy, when possible. and then adjuvant chemotherapy. Age is an important consideration in the treatment of GBM, as it is a negative prognostic indicator. Therefore, the purpose of this review was to explore the benefits and harms of radiotherapy in elderly people with GBM, summarize current evidence for the incremental resource use, utilities, costs, and cost-effectiveness associated with radiotherapy, and provide meaningful conclusions for clinical practice and further research.

## 4 Strengths and limitations of this study

To the best of our knowledge, this will be the first systematic review investigating radiotherapy for the treatment of elderly people with GBM. Currently, there is no clear consensus on how to apply the available evidence to guide treatment of the individual person seen in clinic. Our study will help to inform the best approach to the treatment of older individuals with GBM and help to identify research gaps.

Limitations mainly included the number of studies meeting our screening criteria could be limited; due to language barriers, only 2 languages of the trials can be included, other related studies may be missing.

## Author contributions

All authors critically revised the article for important intellectual content and approved the final version of the manuscript.

**PX Huang:** planned and designed the research

**PX Huang and LQ Li:** project development, data collection, analysis and interpretation, manuscript writing, article revised

**JT Qiao and X Li:** data collection, analysis and interpretation,

**X Li:** data analysis and interpretation

**LQ Li:** data collection, article revised

**P Zhang:** academic oversight and edited all drafts

**Conceptualization:** Xiang Li.

**Data curation:** Liqiang Li, Juntang Qiao.

**Formal analysis:** Juntang Qiao.

**Funding acquisition:** peng Zhang.

**Investigation:** Liqiang Li, Xiang Li.

**Methodology:** Puxin Huang, Xiang Li.

**Project administration:** Puxin Huang, Xiang Li.

**Resources:** Liqiang Li, Xiang Li.

**Software:** Liqiang Li, Juntang Qiao.

**Supervision:** peng Zhang.

**Writing – original draft:** Puxin Huang.

**Writing – review & editing:** Puxin Huang, peng Zhang.

## Supplementary Material

Supplemental Digital Content
