# Supplementary material for: Radiotherapy for glioblastoma in the elderly: A protocol for systematic review and meta-analysis
Source: Medicine (Baltimore). 2020 Dec 24;99(52):e23890. doi: 10.1097/MD.0000000000023890 (PMC7769296; doi:10.1097/MD.0000000000023890)
Supplement: Supplemental Digital Content [file medi-99-e23890-s001.docx]

**The specific search strategy will be (taking PubMed as an example):**

1. Glioblastoma[mh]

2. (glioblastoma* or Glioblastoma* or GB* or astrocyt*). ti,ab.

3. 1 or 2

4. Aged [mh]

5. (aged* or old* or ageing* or geriatric*). ti,ab.

6. (elder* or "over 70" or "70 year*”). ti,ab.

7. 4 or 5 or 6

8. 3 and 7

9 Radiotherapy

10 (radiotherap$ or radiat$ or irradiat$ or teletherap$ or proton$ or plaque). ti,ab.

11. 9 or 10

12. randomized controlled trial.pt.

13. controlled clinical trial.pt.

14. randomized.ab.

15. placebo.ab.

16. clinical trials as topic.sh.

17. randomly.ab.

18. trial.ti

19. 12 or 13 or 14 or 15 or 16 or 17 or 18

20. (animals not (humans and animals)).sh.

21. 19 not 20

22. 8 and 11

23. 21 and 22
